# Status of Pulmonary Metastasectomy After PuLMiCC Trial: A Survey Amongst Oncologists, Gynecologists, Urologists and Dermatologists on Medical Needs for Local Therapy

**DOI:** 10.3390/cancers17243959

**Published:** 2025-12-11

**Authors:** Daniel Baum, Markus Grafe, Rahel Decker, Lysann Rostock, Andreas Friedrich, Till Plönes

**Affiliations:** 1 Department of Thoracic Surgery, Fachkrankenhaus Coswig, Neucoswiger Str. 21, D-01640 Coswig, Germany; 2 Division of Thoracic Surgery, Department of Visceral, Thoracic and Vascular Surgery, Faculty of Medicine, University Hospital Carl Gustav Carus, Technische Universität Dresden, D-01307 Dresden, Germany; 3 National Center for Tumor Diseases (NCT/UCC), German Cancer Research Center (DKFZ), Faculty of Medicine, University Hospital Carl Gustav Carus, Technische Universität Dresden, Helmholtz-Zentrum Dresden—Rossendorf (HZDR), D-01307 Dresden, Germany

**Keywords:** pulmonary metastasectomy, local ablative therapy, oligometastatic disease, interdisciplinary survey

## Abstract

Background: The role of pulmonary metastasectomy has been increasingly questioned in the surgical community after the PulMiCC trial challenged its benefit in colorectal cancer. However, the view on pulmonary metastasectomy among people in non-surgical disciplines remains unclear. This study explored interdisciplinary attitudes toward pulmonary metastasectomy and identified the clinical expectations shaping its future role. Methods: An anonymous online survey of active board-certified physicians in oncology, urology, gynecology and dermatology was conducted (December 2024–June 2025). Twenty items covered attitudes to local ablative therapy, referral criteria, preferred modalities and future relevance. Group comparisons used Pearson’s χ^2^; ordinal ratings were compared by one-way ANOVA; associations were explored with Spearman’s ρ. Results: Of 2884 contacted physicians, 165 participated (≈5.7%), and 106 completed the questionnaire. All 106 (100%) endorsed local ablative therapy as meaningful; 92/106 (86.8%) favored routine integration into multimodal care. Surgical metastasectomy was selected by 49/106 (46.2%), SBRT was selected by 27/106 (25.5%) and image-guided ablation was selected by 7/106 (6.6%); preference for surgery differed by specialty (χ^2^(4) = 15.31, *p* = 0.004), while institutional availability (in-house thoracic surgery or radiation oncology) showed no association with selecting surgery or SBRT. Key referral determinants were number of lesions (105/106; 99.1%), anatomical location (86/106; 81.1%; *p* < 0.02 across specialties), and lesion size (81/106; 76.4%; *p* < 0.05); other factors showed no consistent inter-specialty differences. The perceived usefulness of metastasectomy was high (mode 8/10) and showed a weak, non-significant correlation with referral experience (ρ = 0.172, *p* = 0.077). Looking ahead, 46/106 (43.4%) anticipated a declining role of local ablative therapy with novel systemic therapies; interest in biomarker analysis from metastatic tissue compared to primary tumor tissue was very high 97/106 (91.5%). Conclusions: Local ablative therapy, particularly pulmonary metastasectomy, continues to be viewed as an integral and trusted element of metastatic disease management across specialties. Despite limited prospective evidence, clinicians maintain strong confidence in its clinical value and foresee its evolution toward biologically and patient-tailored indications. However, the interpretation of these findings is limited by a low response rate and potential selection bias toward European, academically affiliated respondents. To our knowledge, this is the first study to systematically capture perceptions of pulmonary metastasectomy among non-surgical oncology-related specialists.

## Simple Summary

Pulmonary metastasectomy, the surgical removal of lung metastases, has long been used alongside systemic treatments, but its benefit is debated. We surveyed physicians from oncology, urology, gynecology and dermatology to learn how they view local treatments for metastatic disease and to identify the factors that guide referrals. All respondents considered local therapy to be meaningful, and nearly nine in ten supported its routine use within multimodal care. The number, size and anatomical location of metastases were the most important referral factors. Many clinicians endorsed surgical removal, others favored stereotactic body radiotherapy and most preferred an individualized approach. Usefulness ratings for metastasectomy were high. Interest in analyzing biomarkers from metastatic tissue was also very high. These findings can inform future studies and support clearer, evidence-based selection of patients for local treatment.

## 1. Introduction

The introduction of pulmonary metastasectomy has its roots in the era before most systemic therapies were implemented in clinical practice and gained a well-established place in thoracic surgery [1]. Pulmonary metastases may arise either synchronously at initial diagnosis or metachronously during follow-up, and clinical decision making often differs between these scenarios. Over recent decades, pulmonary metastasectomy has evolved from an experimental approach to a standard local therapy for selected patients with limited metastatic burden, supported largely by retrospective data rather than prospective trials [2]. Although pulmonary metastasectomy is very common and often performed [3], there is a controversial, ongoing discussion about its effectiveness [4,5,6,7,8]. This controversy stems mainly from the absence of high-level evidence demonstrating a clear clinical advantage, as well as from the heterogeneity of the underlying primary tumors and patient populations [9]. In general, it is an intricate task to conduct a trial investigating active treatment versus taking a watch-and-wait approach in a fatal disease; this seems to be even more complicated in the case of pulmonary metastasectomy, as it is not known whether the procedure itself has the same impact in different disease (e.g., sarcoma, gastric cancer, colorectal carcinoma, etc.). Ethical and logistical barriers further complicate randomized controlled trials in this setting, where equipoise is often difficult to achieve and both patients and physicians may have strong preferences for intervention [10]. In addition, it is questionable which endpoints could truly demonstrate the advantages of pulmonary metastasectomy, and which benefits can be expected, according to the treating physicians, throughout the course of different diseases. Besides overall survival (OS), being one of the strongest clinical endpoints, potential benefits such as improved local control, delayed systemic progression, or better quality of life might also play relevant roles but are rarely addressed in existing studies [11]. The indication for pulmonary metastasectomy can vary from case to case depending on the current situation of the patient and even extend beyond simply prolonging OS. Many thoracic surgeons are firmly convinced that pulmonary metastasectomy improves disease control and extends patient survival, but there is no clear evidence to prove this [7,8]. This belief persists despite the fact that available data remain largely retrospective and are prone to selection bias [4]. The randomized multicenter trial “Pulmonary Metastasectomy versus Continued Active Monitoring in Colorectal Cancer (PulMiCC)” addresses this question, at least for colorectal metastases, but leaves room for more open questions [8]. While the PulMiCC trial did not demonstrate a significant benefit for surgical intervention, its limited sample size and premature termination have prompted ongoing debate within the thoracic surgery community [6]; surgeons in this group seem to be clearly biased regarding pulmonary metastasectomy. We were interested in exploring how other specialties like oncologists, urologists, gynecologists and dermatologists experience every-day clinical practices and local therapies, and what they think about this topic. Previous surveys, such as the recent European Society of Thoracic Surgeons (ESTS) study, have shown that most thoracic surgeons still strongly support pulmonary metastasectomy despite limited prospective evidence [7]. However, an additional and largely overlooked evidence gap concerns the perceptions and referral behaviors of non-surgical oncology-related specialties. Although these disciplines manage most patients with metastatic disease, virtually no data exist on how they assess the value of pulmonary metastasectomy or which criteria guide their referral decisions. We aimed to identify the possible benefits of the treatment beyond the extending OS, and wanted to determine whether other specialties see any value in pulmonary metastasectomy. Understanding whether non-surgical disciplines share this perspective is critical, as oncologists, gynecologists, urologists and dermatologists often serve as the primary decision-makers or referrers for patients who are eligible for local therapies.

Importantly, in routine clinical practice, patients with metastatic disease are usually managed by organ-specific disciplines such as oncology, urology, gynecology and dermatology. These specialists act as gatekeepers for local ablative procedures: they initiate staging, counsel patients and decide whether referral for surgical evaluation is appropriate.

Their views on the disease and its treatment may be different, and the possible answers may lead to more clinically oriented endpoints for future studies. Therefore, we conducted an explorative survey among active oncologists, urologists, gynecologists and dermatologists. By comparing their responses, we aim to identify differences in treatment philosophies, expectations and attitudes towards pulmonary metastasectomy across specialties and geographic regions.

## 2. Materials and Methods

We identified board-certified specialists in the fields of oncology, urology, gynecology and dermatology. Potential participants were identified through institutional websites, publicly available directories and professional networking platforms. This approach ensured the reproducibility of the sampling frame while acknowledging that online-based identification inherently favors individuals with publicly accessible professional profiles. A total of 2884 board-certified specialists were invited to participate in the survey by email between December 2024 and February 2025. The questionnaire was available for completion from December 2024 through to February 2025 using a commercially available platform (www.surveymonkey.com). Participants represented twelve countries: Germany, Austria, Switzerland, France, United Kingdom, Spain, Italy, Belgium, United States, China, Argentia and Brazil. During the period December 2024–June 2025, three reminder emails were sent to boost responsiveness. All participants were informed that the survey was completely anonymous and participation was voluntary. No identifiable data were collected at any stage, and IP addresses were not stored, ensuring full compliance with European data protection regulations.

Survey design: The survey contained twenty questions, which were structured in four sections. In the first section of the survey, we asked for general demographic data, which included age, gender and the geographic location of the respondents’ practices. In the second section, we collected data regarding the respondents’ working environments, e.g., academic environment, and on whether their working environments provide access to local therapy services. In the third section, we investigated attitudes towards pulmonary metastasectomy. In the last section, we asked in more detail about cancer-specific treatments and sought to determine how the role of pulmonary metastasectomy fits in current treatment regimes. We were not interested in surgical details or optimized techniques, but were instead interested in exploring the possible value of pulmonary metastasectomy from the points of view of different specialties. A pilot test involving a small group of physicians (n = 5) was performed to ensure the clarity, content validity and length acceptability of the questionnaire. No major issues regarding comprehensibility or perceived burden were reported. The questions were carefully worded to avoid any bias.

The full questionnaire is provided in Supplementary Material S1.

Data collection: Participants were informed via email about the survey purpose and data privacy conditions.

The ethics committee of the Saxony Medical Association, citing Article 15(1) of the Saxony Code of Ethics regarding research requiring ethical review, confirmed that the study did not require ethical approval because it did not involve patients and did not contain any patient-identifiable data.

Statistical analyses: Categorical variables were summarized as absolute and relative frequencies (n, %) and compared across medical specialties using the Pearson’s χ^2^ test. For items allowing multiple responses, each item was analyzed as an independent binary variable. Ordinal variables (e.g., age or usefulness ratings) were presented as median and range and compared between groups using one-way ANOVA. Correlations between ordinal and continuous variables were assessed using Spearman’s rank correlation coefficient (ρ). All statistical analyses were performed using MedCalc^®^ Statistical Software version 20.104 (MedCalc Software Ltd., Ostend, Belgium) and JASP^®^ (Version 0.95.4; JASP Team, University of Amsterdam, Amsterdam, The Netherlands). A *p*-value < 0.05 was considered statistically significant.

## 3. Results

### 3.1. Respondent Characteristics

We contacted 2884 physicians; 165 participated, among whom 106 completed the questionnaire in full. Most respondents were based in Europe.

The majority of respondents were urologists (n = 50; 47%), followed by oncologists (n = 23; 22%), gynecologists (n = 14; 13%) and dermatologists (n = 10; 9%). The control group consisted of a limited group of thoracic surgeons (n = 9; 8.5%). Most participants (n = 77; 73%) were affiliated with a university hospital, while 29 (27%) worked in non-academic hospitals or private practices. Of the participants, 50% were men, and 50% were women, with a median age of 45 years (range: 29–70 years). The median time since board certification was 10.4 years (range: 0–39 years), which reflects a broad clinical working experience. A summary of respondent characteristics is provided in Table 1.

### 3.2. General Attitudes Toward Local Ablative Therapy

**Figure 1:**
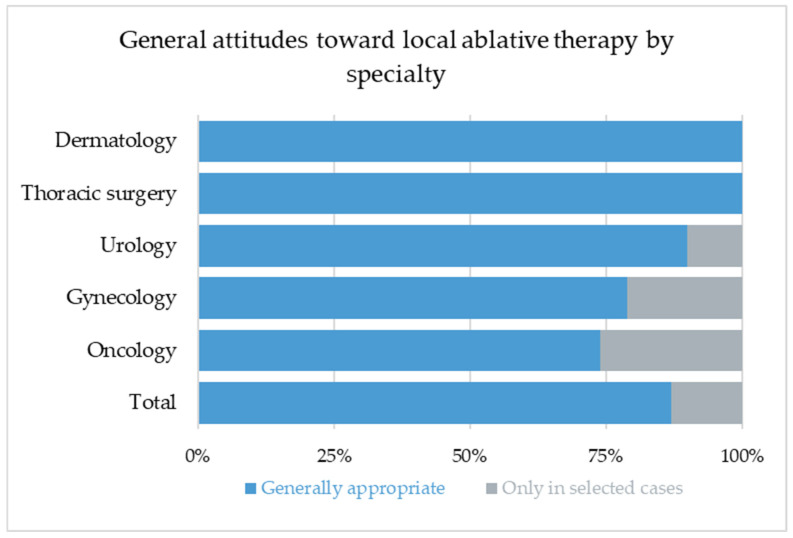
Distribution of responses by specialty regarding the general appropriateness of local ablative therapy in the management of metastatic disease. Each bar represents the proportion of physicians within a specialty who considered local therapy “generally appropriate” (blue) or “appropriate only in selected cases” (light gray). No respondents rejected local therapy. Differences between specialties were not statistically significant (χ^2^ = 7.494, df = 4, *p* = 0.112).

All (106/106; 100%) respondents considered local ablative treatment to be a meaningful component in the management of metastatic malignancies. Among them, 92/106 participants (86.8%) indicated that local therapy should generally be integrated into multimodal treatment concepts, while 14/106 (13.2%) regarded it as appropriate only in selected individual cases. No respondent rejected the use of local therapy completely. By specialty, full endorsement (“generally appropriate”) rates were 100% for dermatology 10/10 (100%) and thoracic surgery 9/9 (100%); for urology, the rate was 45/50 (90%), for gynecology, the rate was 11/14 (78.6%) and for oncology, the rate was 17/23 (73.9%). Differences across specialties were not statistically significant (χ^2^ = 7.494, df = 4, *p* = 0.112). A 100% stacked bar chart is provided in Figure 1.

### 3.3. Indication Criteria for Referral to Local Therapy

**Figure 2:**
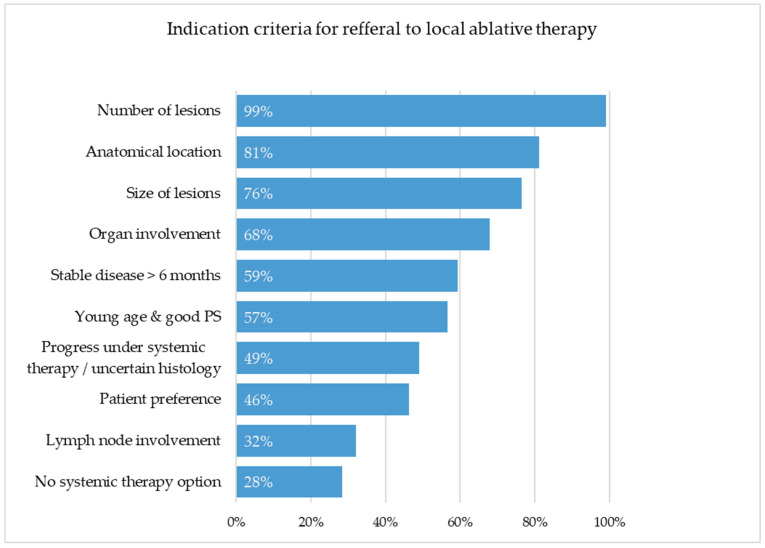
Relevance ratings of clinical and patient-related criteria influencing referrals for local ablative treatment of pulmonary metastases. The number of lesions (99.1%) was considered to be the most important factor, followed by anatomical location (81.1%) and lesion size (76.4%). Disease stability (59.4%) and patient preference (46.2%) were less frequently selected, while the absence of systemic therapy options (28.3%) and lymph node involvement (32.1%) were least relevant.

Respondents were asked to evaluate several clinical and patient-related factors influencing referral for local ablative treatment of pulmonary metastases. The results are summarized in Figure 2.

#### 3.3.1. Lesion-Related Factors

Among all participants, the following findings were observed: The number of lesions was considered a relevant factor by 105/106 (99.1%) of respondents, without significant inter-specialty differences (χ^2^ = 6.634, df = 4, *p* = 0.157). The size of lesions was rated relevant by 81/106 (76.4%) of participants, showing significant differences across specialties (χ^2^ = 9.750, df = 4, *p* = 0.045). Urologists most frequently considered lesion size relevant (42/50 [84%]), followed by oncologists (19/23 [83%]) and thoracic surgeons (5/9 [56%]), whereas dermatologists (7/10) and gynecologists (10/14) were less likely to rate it as relevant (both 70%). The anatomical location of lesions was judged to be relevant by 86/106 (81.1%) of respondents; here, there was also significant variation between specialties (χ^2^ = 11.950, df = 4, *p* = 0.018). Urologists (43/50 [86%]) and oncologists (21/23 [91%]) more often emphasized anatomical location, compared to gynecologists (8/14 [57%]) and thoracic surgeons (5/9 [56%]).

#### 3.3.2. Disease Context and Alternatives

Disease stability (>6 months) was considered important by 63/106 (59.4%) of respondents, without significant differences (χ^2^ = 4.983, df = 4, *p* = 0.289). The unfeasibility of systemic therapy options was viewed as relevant by 30/106 (28.3%), with significant variation between specialties (χ^2^ = 10.040, df = 4, *p* = 0.040). Dermatologists (6/10; 60%) and thoracic surgeons (5/9; 56%) rated this criterion as relevant more often than oncologists (5/23; 22%) and urologists (11/50; 22%). The organ site of metastases (affected organ) was rated as relevant by 72/106 (67.9%) of respondents, with significant heterogeneity between specialties (χ^2^ = 16.860, df = 4, *p* = 0.002). Urologists (30/50; 60%) and oncologists (20/23; 87%) most frequently regarded organ involvement to be relevant, whereas thoracic surgeons (2/9; 22%) and dermatologists (1/10; 10%) did so less often. Lymph node involvement was regarded as relevant by 34/106 (32%) of respondents, without significant differences (χ^2^ = 5.183, df = 4, *p* = 0.269).

#### 3.3.3. Patient-Related Considerations

Patient preference was considered relevant by (49/106; 46.2%), showing no significant inter-specialty difference (χ^2^ = 3.311, df = 4, *p* = 0.507). Progression under systemic therapy combined with histologic uncertainty was judged to be relevant by 52/106 (49.1%) of respondents, showing significant inter-specialty differences (χ^2^ = 11.760, df = 4, *p* = 0.019). Dermatologists (8/10; 80%) and thoracic surgeons (6/9; 67%) reported this factor to be relevant more frequently than urologists (18/50; 36%). Young patient age and good performance status were considered relevant by 60/106 (56.6%) of respondents, without significant differences between specialties (χ^2^ = 7.183, df = 4, *p* = 0.127).

### 3.4. Preferred Local Treatment Modalities

**Figure 3:**
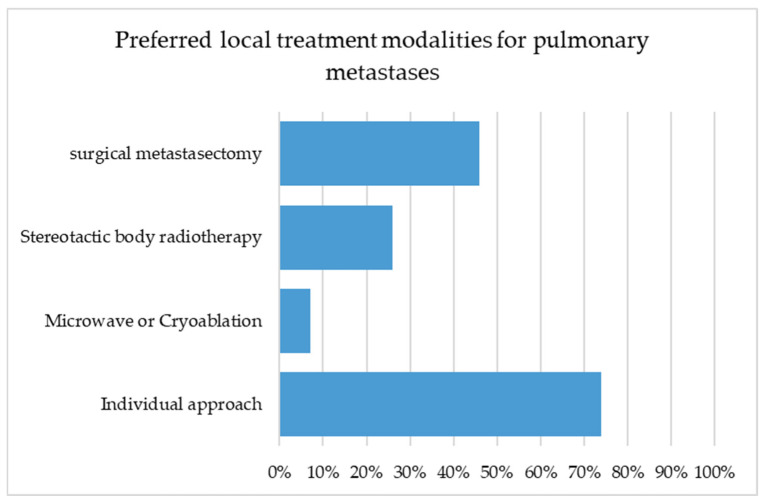
Distribution of responses regarding preferred local treatment modalities for pulmonary metastases. Multiple selections were allowed. Surgical metastasectomy was chosen by 46.2% of respondents, an individualized approach by 74.5%, stereotactic body radiotherapy (SBRT) by 25.5%, and thermal ablation (microwave or cryo) by 6.6%.

There was a question regarding preferred local therapeutic modalities in the management of metastatic disease. Multiple answers were allowed. Overall, surgical metastasectomy was selected by 49/106 (46.2%) as a suitable local therapy. When stratified by specialty, the proportion of respondents favoring surgery differed significantly (χ^2^(4) = 15.31; *p* = 0.004). The highest proportion was observed among thoracic surgeons (8/9; 88.9%), followed by urologists (27/50; 54.0%), oncologists (9/23; 39.1%), gynecologists (4/14; 28.6%), and dermatologists (1/10; 10.0%). Stereotactic body radiotherapy (SBRT) was considered appropriate by 27/106 (25.5%) of respondents, with no statistically significant variation between specialties (χ^2^(4) = 4.95; *p* = 0.292). An individualized approach (depending on patient condition, lesion characteristics, and treatment burden) was endorsed by 79/106 (74.5%) of participants, without significant inter-specialty differences (χ^2^(4) = 7.34; *p* = 0.119). Microwave or cryoablation treatment was selected by 7/106 (6.6%) of respondents, also without significant differences between specialties (χ^2^(4) = 7.87; *p* = 0.096). The reported preferences are summarized in Figure 3.

### 3.5. Institutional Availability and Treatment Choice

The availability of a thoracic surgeon within the respondents’ institution was not associated with selecting surgical metastasectomy as a preferred local treatment option (χ^2^(1) = 1.39, *p* = 0.239; Fisher’s exact test *p* = 1.000). Similarly, the presence of an in-house radiation oncology department or a fixed cooperation partner showed no significant relationship with the use of stereotactic body radiotherapy (SBRT) (χ^2^(1) = 0.35, *p* = 0.557; Fisher’s exact test *p* = 1.000).

### 3.6. Experience with Pulmonal Metastasectomy and Perceived Usefulness

**Figure 4:**
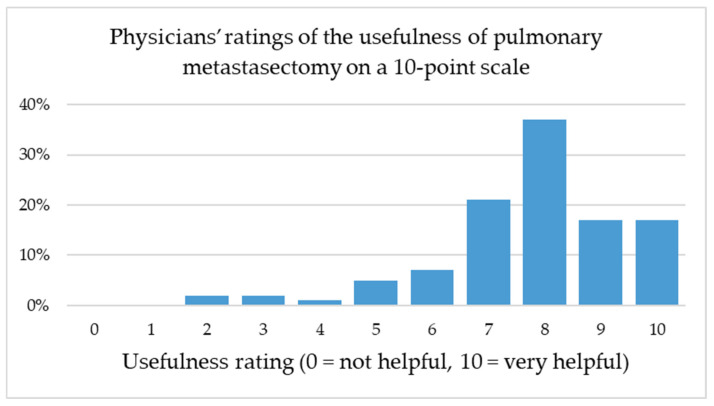
Distribution of responses to the question “How helpful do you consider pulmonary metastasectomy in the management of metastatic disease?” Ratings ranged from 0 (“not helpful”) to 10 (“very helpful”), with a mode of 8. No statistically significant differences between specialties were observed (ANOVA: F(4, 101) = 2.10, *p* = 0.087).

A significant association was observed between medical specialty and the number of patients referred for pulmonary metastasectomy (χ^2^(12) = 43.98, *p* < 0.001). Thoracic surgeons and urologists reported markedly higher referral experience, with most respondents indicating more than 50 or even more than 100 referred patients. In contrast, dermatologists and gynecologists predominantly reported limited experience (1–10 or 10–50 patients), while oncologists were mainly distributed across intermediate categories. Ratings of the perceived usefulness of pulmonary metastasectomy on a 10-point scale (1 = not helpful, 10 = very helpful) ranged from 2 to 10, with a mode of 8 (see Figure 4). A one-way ANOVA comparing medical specialties (dermatology, gynecology, hematology/oncology, thoracic surgery and urology) revealed no statistically significant differences: F(4, 101) = 2.10, *p* = 0.087, η^2^ ≈ 0.08. A weak positive correlation was observed between the number of patients referred for pulmonary metastasectomy and the perceived usefulness of the procedure (Spearman’s ρ = 0.172, *p* = 0.077). This trend did not reach statistical significance.

### 3.7. Perceived Risks of Pulmonary Metastasectomy

In response to the question “Which disadvantage do you most fear in patients undergoing pulmonary metastasectomy?”, 27/106 (25.5%) of participants stated that they do not fear any disadvantages. Among those who indicated potential risks, the most frequently mentioned concerns were dyspnea (18/106; 17%), pain (15/106; 14%), a high risk of complications (15/106; 14%), loss of quality-of-life (QoL) (14/106; 13%), and temporary ineligibility for (chemo)therapy (13/106; 12%). Only 2/106 (1.9%) of respondents mentioned the absence of a radiologic correlate for treatment monitoring as a disadvantage.

### 3.8. Anticipated Future Relevance of Local Therapy

**Figure 5:**
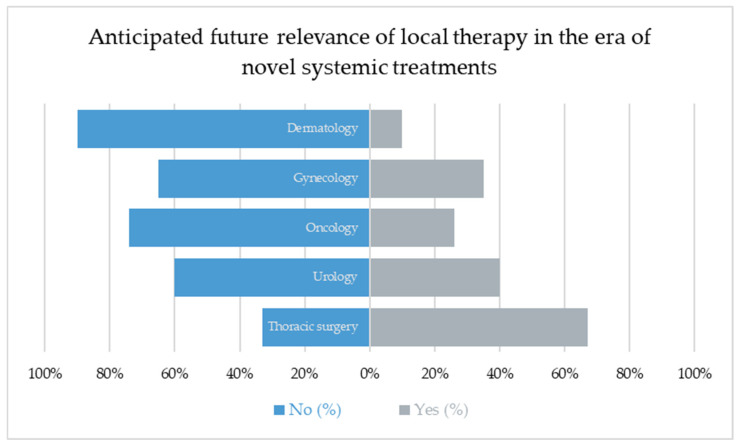
A significant inter-specialty difference was observed (χ^2^(4, N = 106) = 14.23, *p* = 0.007). Thoracic surgeons most frequently expected a declining role (67%), whereas dermatologists (10%) and oncologists (26%) were the least likely to share this view.

A significant difference between specialties was found regarding the anticipated future relevance of local therapies in the context of novel systemic treatments (χ^2^(4, N = 106) = 14.23, *p* = 0.007; see Figure 5). Thoracic surgeons were the most likely to expect a declining role of local interventions (6/9; 67%), whereas dermatologists (1/10; 10%) and oncologists (6/23; 26%) were least likely to share this view. Overall, 46/106 (43%) of respondents anticipated a future decrease in the importance of local ablative therapy in the context of novel systemic treatments.

### 3.9. Interest in Biomarker Analysis from Metastatic Tissue

**Figure 6:**
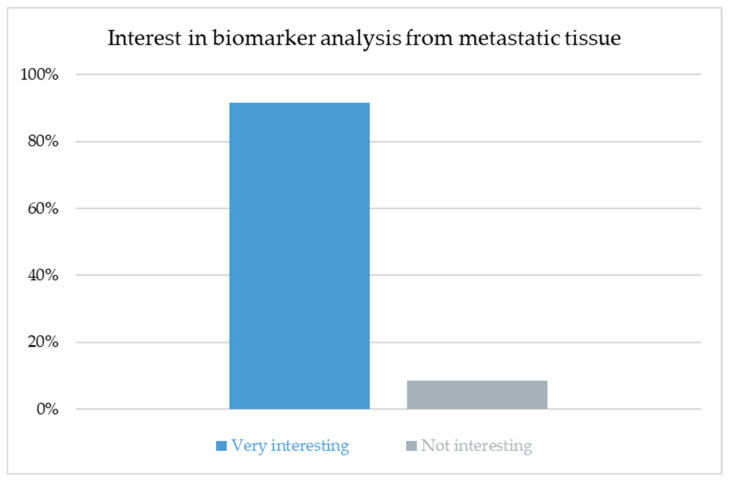
Nearly all respondents (91.5%) rated the analysis of biomarkers from metastatic tissue as highly interesting, with no significant difference across specialties.

Among all respondents, 97/106 (91.5%) rated the analysis of biomarkers from metastatic tissue in the course of treatment to adapt systemic therapy as very interesting, while 9/106 (8.5%) considered it not interesting (see Figure 6). The proportion of respondents rating the topic as “very interesting” was 8/10 (80%) among dermatologists, 14/14 (100%) among gynecologists, 22/23 (95.7%) among oncologists, 8/9 (88.9%) among thoracic surgeons, and 45/50 (90%) among urologists. The association between specialty and level of interest was not statistically significant (χ^2^(4) = 3.738; *p* = 0.443).

## 4. Discussion

Pulmonary metastasectomy is still discussed with controversy and is a matter of debate [12]. The majority of thoracic surgeons perform pulmonary metastasectomy to some extent [3,7,13,14,15], but conviction that this will improve OS and disease control is widespread. Despite several methodological and technical aspects that have been widely debated, the PulMiCC trial delivered a message that many thoracic surgeons may find difficult to accept, as it challenges long-held assumptions regarding the survival benefit of pulmonary metastasectomy. On the other hand, there are data (even if they are of a retrospective nature) which indicate that metastasectomy may be beneficial; it is also plausible that local therapies could work in the field of oligometastatic lung cancer (with much broader evidence) [16,17,18,19]; moreover, different techniques like stereotactic body radiotherapy also have more evidence supporting them [20,21,22,23].

This leads to the question of whether most thoracic surgeons just ignore the evidence or whether the PulMiCC trial did not tell the whole story. Interestingly, oncologists seem to be open or even convinced that the addition of local therapies may be beneficial, even if they should be less biased than surgeons toward surgical therapies. Our survey confirms a generally positive perception of local therapy across disciplines. Remarkably, all respondents (100%) expressed openness toward the concept of pulmonary metastasectomy, and 87% considered local treatment to be an integral component of the multimodal management of metastatic disease. The remaining participants regarded it as appropriate at least in selected individual cases, while none rejected local therapy completely. This finding indicates a striking level of consensus that transcends specialty boundaries and suggests that, despite the lack of high-level evidence, local ablative approaches continue to hold a strong position in modern oncology.

Such universal endorsement likely reflects the shared clinical perception that local disease control can contribute meaningfully to overall patient management, whether by reducing tumor burden, delaying systemic progression, or improving symptom control. It may also represent a degree of therapeutic optimism that persists despite ongoing controversy about the true survival benefits of pulmonary metastasectomy.

Unlike previous surveys focusing on thoracic surgeons, our study deliberately targeted non-surgical specialists who function as primary decision-makers and gatekeepers in metastatic disease management. Their referral decisions based on tumor biology, systemic treatment plans and patient-specific considerations determine whether surgical evaluation is pursued. Understanding these perspectives is therefore critical for a comprehensive picture of real-world practice. While formal indications and contraindications for pulmonary metastasectomy, SBRT or thermal ablation must be determined by thoracic surgeons or radiation oncologists, referral pathways are shaped by organ-specific specialists who manage metastatic patients in routine practice. Their preferences reflect clinical experience, institutional collaborations, perceived treatment value and the limited high-level evidence for all local ablative modalities. Technical decisions regarding the choice of wedge resection versus ablative procedures, however, remain within the expertise of thoracic surgeons or radiation oncologists and were not evaluated by this survey. Our survey therefore captures subjective referral tendencies rather than procedural eligibility, which remains the responsibility of the respective specialist.

Although all respondents consider local therapy to be an essential component in the management of metastatic disease, only 46% regarded pulmonary metastasectomy as the treatment of choice, while 75% favored an individualized approach and 25% considered SBRT to be the most appropriate modality. It is not surprising that thoracic surgeons and urologists showed the highest preference for surgical resection, as these groups are most frequently involved in surgical treatment of metastases.

No significant institutional bias could be demonstrated regarding the preferred type of local therapy. These findings suggest that therapeutic preferences were not primarily determined by institutional infrastructure but rather by individual or specialty-specific perspectives.

When evaluating factors influencing referral for local ablative treatment of pulmonary metastases, respondents showed both consensus and marked inter-specialty heterogeneity.

The number of lesions was almost unanimously considered to be relevant (99%), while lesion size (*p* = 0.045) and anatomical location (*p* = 0.018) were assessed differently across disciplines. Urologists and oncologists emphasized these criteria more often, whereas thoracic surgeons and gynecologists rated them as less decisive, possibly because surgeons view resectability as a given once surgical indication is discussed. Surprisingly, lymph node involvement (32%) was not considered an important factor, despite clear evidence of its negative prognostic impact [24,25] and the fact that about 70% of thoracic surgeons regard it as a contraindication to metastasectomy [7].

Notably, the perceived benefit of pulmonary metastasectomy was rated highly across all specialties, with a mode of 8 on a 0–10-point scale even among non-thoracic disciplines. This finding underlines the broad interdisciplinary recognition of pulmonary metastasectomy as a valuable therapeutic option, despite ongoing debate regarding its evidence base and indication spectrum. A weak positive correlation was observed between the number of patients referred for pulmonary metastasectomy and the perceived usefulness of the procedure (Spearman’s ρ = 0.172, *p* = 0.077), suggesting that greater clinical experience may be associated with a more favorable perception, although this trend did not reach statistical significance.

In response to the question “Which disadvantage do you most fear in patients undergoing pulmonary metastasectomy?”, 26.0% of participants stated that they do not expect any disadvantages, which is remarkable given the invasive nature of the procedure.

Among those who reported potential risks, the most frequently mentioned concerns were dyspnea (17.3%), pain (14.4%), a high risk of complications (14.4%), loss of quality of life (13.5%), and temporary ineligibility for systemic therapy (12.5%). These findings indicate that concerns about postoperative morbidity and functional loss are comparatively infrequent among respondents. Indeed, Baum et al. demonstrated that, after single-laser-assisted metastasectomy, no significant long-term decline in lung function occurs [26].

The high level of interest in biomarker analysis across all specialties (91.5%) underscores the growing recognition that the molecular profiling of metastatic tissue will become integral to individualized treatment planning. Biomarker-driven strategies have already reshaped systemic oncology and are likely to influence decisions regarding local ablative therapy as well. Future concepts of pulmonary metastasectomy may increasingly combine morphologic and molecular criteria, identifying patients who derive the most benefit from surgical intervention. Integrating histo-molecular data into multidisciplinary tumor boards could refine indications, optimize timing and improve patient selection for local therapy.

Despite major advances in systemic oncology, including immunotherapy and targeted agents, most respondents did not anticipate the diminishing role of local ablative treatments. Overall, 43% expected a future decrease in importance, while the majority envisioned that local therapies would remain integral to multimodal concepts.

Notably, thoracic surgeons (67%) were most likely to anticipate a declining role, perhaps reflecting a self-critical awareness of the limited high-level evidence, whereas dermatologists (10%) and oncologists (26%) were least likely to share this view.

These findings underline that confidence in local therapy remains strong, particularly among non-surgical disciplines, suggesting that pulmonary metastasectomy and other ablative approaches will continue to evolve alongside systemic innovations. Rather than being replaced, local treatment is increasingly being perceived as a precision tool that should be applied selectively in well-defined contexts.

The future role of pulmonary metastasectomy will likely evolve alongside advances in systemic oncology. The use of immunotherapy, targeted treatments and emerging biomarkers, such as ctDNA dynamics or metastasis-specific molecular signatures, may help in identifying patients with biologically limited disease who are most likely to benefit from local control. Hybrid treatment concepts that integrate systemic therapy with selective use of surgery or SBRT are increasingly being explored, particularly for oligometastatic or oligoprogressive disease, as illustrated by studies in NSCLC and other solid tumors that combine systemic treatment with local consolidative or oligoprogression-directed radiotherapy [16,17,19,27]. The strong interest in biomarker analysis observed in our survey reflects growing expectations among clinicians that precision oncology frameworks will guide patient selection, refine treatment timing, and potentially reshape indications for local ablative therapy.

The observed heterogeneity between specialties may also reflect discipline-specific belief systems and cultural practice patterns. For example, oncologists increasingly rely on highly effective systemic therapies and may therefore value local treatment primarily as a complementary option, whereas surgical disciplines traditionally attribute a stronger role to resection-based local control. Dermatologists, gynecologists, and urologists may interpret pulmonary metastases through the lens of their respective tumor entities, systemic treatment algorithms and institutional norms. As modern systemic therapies continue to evolve, particularly immunotherapy and targeted agents, these specialty-specific expectations may further influence referral behavior and perceived benefits of local ablative procedures.

A major limitation of this study is the exceptionally low response rate. Out of approximately 2900 physicians who were contacted, only 165 participated in the survey, corresponding to a response rate of 5.7%. Of these, merely 106 respondents (64.2%) completed the questionnaire in full. Low response rates in physician-targeted surveys are a well-documented and expected phenomenon in survey methodology [28]. Even studies conducted under seemingly optimal conditions, such as national, specialty-specific surveys with established professional networks, repeated reminders, and high topical relevance, rarely achieve substantially higher participation. For example, the NutriOnc national survey among young oncology professionals reported a response rate of 20.8% despite direct institutional contact and inherent topic relevance [29]. Several published surveys among medical professionals report response rates between 5–20%, reflecting structural barriers such as email saturation, competing clinical workload and a general decline in survey engagement within the medical community [28]. In this context, the response rate observed in our study is consistent with established patterns and should be interpreted as typical rather than unusually low [30].

In our pilot testing (n = 5), the 20-item questionnaire was not perceived as overly long or complex and, among those who started the survey, the completion rate was relatively high. We therefore consider it more likely that (instead of being attributable questionnaire length or complexity alone) the low response rate reflects the general saturation of physicians’ inboxes with survey invitations and academic requests, as well as the fact that local ablative therapy for pulmonary metastases is not a primary focus for all invited specialties.

Geographic representation was limited, with a predominance of European respondents. Although we aimed to collect data from major regions including Asia, Africa, and South America, we faced substantial practical barriers: reliable contact information for board-certified specialists was often unavailable; English-language invitations may have reduced accessibility; recruitment strategies relying on publicly available institutional websites and professional networking platforms favored physicians with a strong “electronic footprint”. Consequently, important countries such as India and Japan are not represented in our dataset. This geographic and structural skew introduces selection bias and limits the generalizability of our findings. The distribution of specialties and regions was imbalanced, with urologists and European respondents being overrepresented. While this offers detailed insight into a key referrer group, it introduces selection bias and limits the generalizability of our findings. An additional source of potential bias results from the overrepresentation of physicians from academic institutions. These respondents may be generally more receptive to scientific inquiries and more accustomed to participating in research projects.

In an attempt to broaden the reach, we also approached professional medical societies to disseminate the survey among oncologists, gynecologists and urologists in Germany and other European countries. However, these efforts were largely unsuccessful. Remarkably, several gynecological associations declined participation within a very short time, while other societies did not respond at all. This illustrates the difficulty of engaging professional bodies in interdisciplinary research topics that extend beyond their immediate field of focus. Beyond these structural considerations, important evidence gaps remain. Previous research has focused primarily on the perspectives of thoracic surgeons, yet real-world referral decisions are largely shaped by organ-specific specialties such as dermatology, gynecology, urology and oncology. Their views on the appropriateness, timing and perceived benefit of pulmonary metastasectomy had not been systematically investigated before; these groups and their views represent a critical but previously unmeasured determinant of whether surgical evaluation is pursued at all. Although our survey provides initial insight into these gatekeeping dynamics, the cross-sectional design captures attitudes at a single time point and cannot reflect how perceptions may evolve in response to emerging systemic therapies or new clinical evidence. Future research should therefore include longitudinal surveys to monitor temporal changes in referral patterns and belief systems, and comparative studies involving larger thoracic surgery cohorts to map interdisciplinary discrepancies more robustly. Such approaches may ultimately help to refine patient selection and integrate local treatment decisions more effectively into contemporary precision oncology frameworks.

## 5. Conclusions

Pulmonary metastasectomy remains widely accepted across oncological disciplines and is viewed as an integral complement to systemic therapy. Therefore, pulmonary metastasectomy should be seen as a supplement in a multimodal treatment regime rather than an alternative to systemic therapy. While only about half of respondents selected surgery as the preferred local modality and many favored an individualized approach, there was broad agreement on the clinical relevance of local control and a strong interest in biomarker-guided selection. Our findings support an argument for standardized multidisciplinary decision pathways that combine morphologic tumor burden (e.g., number/location of lesions) with histo-molecular profiling to identify those patients most likely to benefit. Given the low response rate and the heterogeneity of views, prospective, biomarker-integrated studies comparing surgery, SBRT and observations are warranted to generate high-level evidence and refine patient selection.

## Figures and Tables

**Table 1:** Baseline characteristics of survey participants: Demographic and professional characteristics of 106 physicians participating in the interdisciplinary survey. The majority of respondents were urologists (47.2%), followed by oncologists (21.7%), gynecologists (13.2%), dermatologists (9.4%) and thoracic surgeons (8.5%). Half of the participants were women. Median age was 45 years (range 29–70), with a median of 10.4 years since board certification. Most respondents worked in university hospitals (73.3%), and the vast majority were based in Europe (93.4%).

Variable	Category	n (%)	Median (Range)
Specialty	Oncology	23 (21.7)	
	Urology	50 (47.2)	
	Gynecology	14 (13.2)	
	Dermatology	10 (9.4)	
	Thoracic surgery	9 (8.5)	
Gender	Men/Women	53 (50.0)/53 (50.0)	
Age (years)			45 (29–70)
Years since board certification			10.4 (0–39)
Institution type	University hospital	77 (73.3)	
	Non-academic hospital/Private practice	29 (26.7)	
Region	Europe	99 (93.4)	
	South America	6 (5.7)	
	Asia	1 (0.9)	
Total		106 (100)	

## Data Availability

The raw data supporting the conclusions of this article will be made available by the authors on request.

## Supplementary Materials

The following supporting information can be downloaded at: https://www.mdpi.com/article/10.3390/cancers17243959/s1, Supplementary Material S1: Interdisciplinary survey on local ablative therapy and pulmonary metastasectomy.

## Abbreviations

The following abbreviations are used in this manuscript:

PulMiCC	Pulmonary Metastasectomy versus Continued Active Monitoring in Colorectal Cancer
OS	Overall Survival
QoL	Quality of Life
SBRT	Stereotactic Body Radiotherapy
ESTS	European Society of Thoracic Surgeons
ANOVA	Analysis of Variance
χ^2^	Chi-squared (Pearson’s chi-square) test
df	Degrees of freedom
ρ	Spearman’s rho
