# Supplementary material for: Status of Pulmonary Metastasectomy After PuLMiCC Trial: A Survey Amongst Oncologists, Gynecologists, Urologists and Dermatologists on Medical Needs for Local Therapy
Source: Cancers (Basel). 2025 Dec 11;17(24):3959. doi: 10.3390/cancers17243959 (PMC12730653; doi:10.3390/cancers17243959)
Supplement: Supplementary file 1 [file cancers-17-03959-s001.zip › cancers-4022219-supplementary.pdf]

cancers

MDPI

Supplementary Material S1 — Survey Instrument (English)

Title: Interdisciplinary survey on local ablative therapy and pulmonary metastasectomy

Target participants: Board-certified physicians in medical oncology/hematology, urology, gynecology, dermatology, and thoracic surgery (control group).

Instructions to participants

• Participation is anonymous and voluntary.

• Please answer based on your routine clinical practice.

• Unless otherwise stated, select one answer per question.

• Where "multiple answers possible" is indicated, select all that apply.

• Definition: Local ablative therapy refers to non-diagnostic local treatments of metastases (e.g., pulmonary metastasectomy, stereotactic body radiotherapy [SBRT], thermal ablation such as microwave or cryoablation).

Section 1 — Demographics & Professional Background

1. Gender

□ Female

□ Male

□ Diverse/Other

□ Prefer not to say

2. Age (years)

□ Open numeric entry

3. Do you work in a university hospital / academic setting?

□ Yes

□ No

4. Year of board certification (primary specialty)

□ YYYY (open entry)

5. Specialty

□ Medical oncology/hematology

□ Urology

□ Gynecology

□ Dermatology

□ Thoracic surgery (control group)

□ Other (free text)

6. Region of practice

□ Europe

□ South America

□ Asia

□ North America

□ Africa

□ Oceania

7. Primary workplace

□ Hospital

 https://doi.org/10.3390/cancers17243959

□ Private practice/Outpatient center
□ Both

Section 2 — General Attitudes to Local Ablative Therapy
8. Do you consider local ablative therapy a meaningful component of care in metastatic malignant disease?
□ Yes, generally appropriate (should usually be integrated into multimodal concepts)
□ Appropriate only in selected individual cases
□ No, not appropriate

Section 3 — Referral Criteria for Local Therapy (Pulmonary Metastases)
For the following items, indicate whether the factor is relevant for referral to a surgeon/radiation oncologist for local therapy.
Response options for each item: □ Relevant □ Not relevant
9. Number of lesions
10. Size of lesions
11. Anatomical location of lesions
12. Stable disease for a prolonged period (>6 months)
13. No feasible systemic therapy option remains
14. Patient's explicit preference for local therapy
15. Organ site(s) affected by metastases (involved organ)
16. Lymph node involvement
17. Progression under systemic therapy combined with histologic uncertainty
18. Young patient age and good performance status

Section 4 — Experience and Perceived Usefulness
19. How many patients with metastases have you referred for pulmonary metastasectomy during your career?
□ 1–10
□ 10–50
□ >50
□ >100
20. How helpful do you consider pulmonary metastasectomy in patient management? (0 = not helpful, 10 = very helpful)
Scale: 0 1 2 3 4 5 6 7 8 9 10

Section 5 — Preferred Local Modality (multiple answers possible)
21. Which local option(s) do you consider most appropriate in principle? (multiple answers possible)
□ Surgical metastasectomy
□ Stereotactic body radiotherapy (SBRT)
□ Thermal ablation (microwave or cryoablation)
□ Individualized approach depending on lesion characteristics, patient condition, and treatment burden
22. Maximum lesion number you would accept to consider pulmonary metastasectomy:
□ Single lesion
□ 1–3
□ 4–5
□ 5–10

□ 10–20
□ >20
□ Not based on a fixed count; depends on technical resectability as judged by the surgeon

Section 6 — Biomarkers and Tumor Entities

23. How interesting/relevant is biomarker analysis from metastatic tissue (e.g., hormone receptors, EGFR, PD-L1) for treatment adaptation?
□ Very interesting
□ Rather interesting
□ Not interesting
24. Which tumor entities do you consider suitable for pulmonary metastasectomy? (multiple answers possible)
□ Colorectal cancer
□ Renal cell carcinoma
□ Breast cancer
□ Lung cancer
□ Osteosarcoma
□ Soft-tissue sarcoma
□ Germ cell tumors
□ Melanoma
□ Ovarian cancer
□ Head-and-neck cancers
□ CUP (cancer of unknown primary)
□ Hepatocellular carcinoma
□ Pancreatic cancer
□ None of the above
25. Which metastatic organ site do you regard as most suitable for metastasectomy? (choose one)
□ Lung
□ Liver
□ Brain
□ Bone
□ Adrenal gland
□ Lymph nodes
□ Metastases reflect systemic disease and are therefore not suitable for organ-focused surgery

Section 7 — Local Infrastructure

26. Is a thoracic surgeon available at your institution or via a fixed cooperation partner?
□ In-house
□ Fixed cooperation partner
□ No
27. Is radiation oncology available at your institution or via a fixed cooperation partner?
□ In-house
□ Fixed cooperation partner
□ No

Section 8 — Outlook & Risk Perception

28. With the advent of novel systemic treatments (e.g., immunotherapy, targeted agents), do you expect the overall importance of local therapy (surgery, SBRT, etc.) to decrease in the future?

□ Yes
□ No
□ Unsure

29. Which disadvantage do you most fear in patients undergoing pulmonary metastasectomy? (choose one)

□ Dyspnea
□ Pain
□ High risk of complications
□ Loss of quality of life
□ Temporary ineligibility for (chemo)therapy
□ No radiologic correlate for treatment monitoring
□ No disadvantages expected
□ Other: ___________

Thank you for your participation.

This instrument may be reused for research with appropriate attribution.
